# A multidomain early warning model for 12-month adverse outcomes in older adults with multimorbidity: a risk-stratified public health approach

**DOI:** 10.3389/fpubh.2026.1841984

**Published:** 2026-09-09

**Authors:** Miao-miao Zhou, Zhong-yao Zhou, Bei-bei Chen, Li-ping Chen, Ying-mei Du, Qingqing Xie

**Affiliations:** 1 Department of Oncology and Radiotherapy, The First Affiliated Hospital of Wenzhou Medical University, Wenzhou, China; 2 Department of Hematology, The First Affiliated Hospital of Wenzhou Medical University, Wenzhou, China; 3 Department of Cardiovascular Medicine, The First Affiliated Hospital of Wenzhou Medical University, Wenzhou, China

**Keywords:** adverse outcomes, early warning model, integrated care, multimorbidity, nomogram, older adults, public health, risk stratification

## Abstract

**Objective:**

To develop and internally validate a multidomain early warning model for 12-month adverse outcomes in older adults with multimorbidity, and to explore its potential for risk-stratified follow-up.

**Methods:**

This retrospective study included 690 older adults (≥60 years) with multimorbidity from a Chinese tertiary hospital, split into derivation (*n* = 483) and validation (*n* = 207) sets. The primary outcome was a composite of unplanned hospitalization, ≥2 emergency visits, new functional decline, or all-cause mortality within 12 months. LASSO and multivariable logistic regression were used for model development. A nomogram was constructed and assessed by discrimination, calibration, and decision curve analysis. Patients were stratified into low, intermediate, and high-risk groups. Multimorbidity network analysis and a retrospective scenario analysis of a model-driven stratified intervention were also performed.

**Results:**

The composite outcome occurred in 146 patients (21.2%). Seven predictors were retained: age ≥75 years, ≥4 chronic diseases, prior hospitalization, ADL impairment, malnutrition, depression, and chronic pain. The model showed good discrimination in the derivation set (AUC 0.847), random split validation set (AUC 0.826), and temporal validation set (AUC 0.819). Bootstrap internal validation yielded an optimism-corrected AUC of 0.831. Calibration was acceptable across all validation methods (calibration slopes: 0.94–1.02; Brier scores: 0.152–0.171). Using pragmatic thresholds, 41.2, 42.0, and 16.8% of patients were classified as low, intermediate, and high risk, respectively. The high-risk group accounted for 41.1% of all events. Network analysis revealed a prominent cardio-metabolic disease cluster. In a retrospective scenario analysis, a model-driven stratified intervention was projected to reduce composite adverse outcomes by 25.3%.

**Conclusion:**

This internally validated model identifies a small high-risk subgroup that carries a large share of adverse events. External validation in community health centers, secondary hospitals, and other regions is required before implementation. The model provides a preliminary framework for targeted follow-up and risk-informed planning, but its generalizability remains unproven.

## 1 Introduction

Population aging has become a defining public health challenge, particularly in health systems facing rapid growth in the number of older adults living with multiple chronic conditions. Multimorbidity is now widely recognized as common in later life rather than an exception, and its rising prevalence has important implications for health service organization, prevention strategies, and long-term care planning (1). Older adults with multimorbidity are more likely to experience disability, poorer quality of life, and greater treatment burden than those with a single chronic disease (2). From a public health perspective, multimorbidity is also associated with increased mortality, adverse drug events, and greater use of health-care resources (3).

The World Health Organization has argued that responses to population aging should move beyond disease-specific management and toward maintaining functional ability across the life course (4). In a similar vein, the WHO Integrated Care for Older People framework emphasizes early identification of declines in intrinsic capacity and the development of person-centered care pathways that can be adapted to local service systems (5). Early identification of declines in these domains allows for targeted interventions to slow or reverse functional loss. A comprehensive review by Zhao et al. (6) on intrinsic capacity found that declines in intrinsic capacity are strongly associated with multiple adverse health outcomes, including mortality, hospitalization, and functional impairment, and that IC serves as an effective predictor of these outcomes. The review also highlighted that intrinsic capacity assessments can be conducted using the ICOPE screening tools, which share substantial overlap with the domains included in our model (6). Within the ICOPE framework, declines in locomotor function (e.g., ADL impairment), vitality (e.g., malnutrition), and psychological status (e.g., depression) are recognized as early markers of reduced intrinsic capacity, and each has been independently associated with adverse health outcomes in older adults.

Current clinical care, however, still frequently follows single-disease models. NICE guidance on multimorbidity has therefore highlighted the need to reduce treatment burden, prioritize outcomes that matter to patients, and prevent uncoordinated or unplanned care (7). One construct that is closely related yet conceptually distinct from multimorbidity is frailty. Frailty is a clinically recognizable state of increased vulnerability resulting from age-related decline in reserve and function across multiple physiologic systems, and it has been consistently shown to predict hospitalization, disability, institutionalization, and mortality in older populations (8). A recent systematic review and meta-analysis by Liu et al. (8) found that multidimensional frailty was a significant predictor of mortality in older adults, with a pooled hazard ratio of 5.48 (95% CI: 3.91–7.67). While multimorbidity refers to the presence of two or more chronic diseases, frailty captures the physiological and functional consequences of accumulated deficits. However, the two often coexist, and their interaction may amplify the risk of adverse outcomes beyond that conferred by disease count alone. Another meta-analysis by Chu et al. (9) systematically summarized the adverse health effects of frailty, demonstrating that frail individuals face substantially elevated risks of mortality, hospitalization, and functional decline across middle-aged and older populations. Notably, several of these domains—functional impairment, malnutrition, depression, and chronic pain—are also central to most frailty instruments, suggesting that pragmatic risk prediction can be achieved without calculating a composite frailty score.

Tinetti et al. (10) similarly argued that multimorbidity should be treated as a central organizing challenge for modern health care rather than as a marginal complication of disease-specific care. Earlier work by Boyd et al. (11) also showed how conventional guideline-driven approaches may become problematic in older patients with multiple conditions, especially when cumulative treatment demands and competing priorities are overlooked. The consequences of multimorbidity are particularly evident in health service utilization. A recent systematic review and meta-analysis found that multimorbidity in older adults was associated with a markedly higher risk of hospitalization and readmission (12). Functional decline is another major concern. A systematic review by Ryan et al. concluded that multimorbidity predicts future functional decline in community-dwelling adults, with stronger effects seen as disease burden increases (13). These findings are important in public health terms because hospital use and functional deterioration often contribute to escalating care needs, caregiver strain, and widening inequities in access to effective long-term management.

Aging itself further intensifies this problem. Fabbri et al. (14) described multimorbidity as a manifestation of age-related loss of resilience and multisystem dysregulation, rather than simply a count of coexisting diseases. This conceptualization helps explain why conventional biomedical indicators alone may not fully capture short-term risk in older patients with chronic disease. In practice, measures of daily function may offer essential complementary information. The Katz Index has long provided a standardized way to assess activities of daily living, and loss of independence in these domains remains one of the clearest markers of vulnerability in older adults (15).

Nutritional and psychological factors are also highly relevant in this population. The Mini Nutritional Assessment Short Form was developed specifically to identify undernutrition risk in geriatric settings and is widely used because of its clinical practicality (16). Depression screening is similarly important, and the PHQ-9 has become one of the most commonly used brief tools for identifying depressive symptom burden in clinical and research settings (17). Together, these domains suggest that a useful early-warning model for older adults with multimorbidity should integrate not only age and disease burden, but also functional, nutritional, and mental health status.

Despite growing recognition of these issues, there remains a gap between conceptual calls for integrated care and the practical ability to identify which older adults are most likely to deteriorate over the near term. Several risk prediction tools exist for older populations, including the Hospital Frailty Risk Score (ICD-10 based) (18), the Probability of Repeated Admission (community screening) (19, 20), and the Multidimensional Prognostic Index (comprehensive geriatric assessment) (21, 22). However, most are designed for specific settings, require specialized assessments, or focus on a single outcome such as mortality. A recent systematic review of multimorbidity prediction models noted significant risks of bias and a lack of models developed specifically for multimorbid populations using routinely available data (23). A composite adverse outcome—including unplanned hospitalization, recurrent emergency visits, functional decline, and mortality—captures the multidimensional deterioration that older adults with multimorbidity often experience as a cascade of interrelated events. Such composites are increasingly recommended for geriatric research because they reflect real-world priorities of patients and health systems, and improve statistical efficiency without focusing on a single pathway (24, 25).

In this context, we developed a pragmatic multidomain early-warning model for older adults with multimorbidity. We focused on routinely available predictors and a composite outcome that reflects real-world priorities. The model was developed and internally validated in a single-center cohort. The simulation analysis of stratified intervention is exploratory and hypothesis-generating, not confirmatory. The findings should be interpreted with caution, and we explicitly identify the steps needed before any clinical or public health application can be considered.

## 2 Methods

### 2.1 Study design and population

This was a retrospective study conducted at The First Affiliated Hospital of Wenzhou Medical University. Patients were included if they were: (1) aged ≥60 years; (2) diagnosed with at least two chronic diseases, such as anemia, diabetes, coronary heart disease, chronic kidney disease, chronic obstructive pulmonary disease, or hypertension (multimorbidity); (3) had complete medical records and at least 12 months of follow-up data. Patients with severe cognitive impairment preventing reliable data collection or with a life expectancy <6 months were excluded.

The study period was from January 2023 to December 2024. For model development and internal validation, the cohort was first randomly split into a derivation set (*n* = 483, 70%) and a random split validation set (*n* = 207, 30%). To further assess temporal stability, we performed a temporal validation using a time-based split: patients enrolled between January 2023 and June 2024 served as the temporal training set (*n* = 518, 75%), and those enrolled between July 2024 and December 2024 served as the temporal validation set (*n* = 172, 25%). The temporal validation set was completely separated from the model development process and used only for final evaluation.

The study was approved by the Institutional Review Board of The First Affiliated Hospital of Wenzhou Medical University, and the requirement for written informed consent was waived due to the retrospective nature of the study. All procedures were performed in accordance with the Declaration of Helsinki.

### 2.2 Data collection and variable definition

Data were extracted from the hospital’s electronic medical record system. All variables were measured at baseline.

Age was recorded as a continuous variable and dichotomized at 75 years or older for analysis, based on the widely used threshold for increased risk in geriatric populations.

The number of chronic diseases was counted using ICD-10 codes from the medical records. We included the following conditions: hypertension, diabetes, heart disease, arthritis, chronic kidney disease, COPD, stroke or transient ischaemic attack, memory-related diseases, and digestive diseases. A count of four or more diseases was used as the cut-off, as this threshold has been associated with higher rates of adverse outcomes in older adults.

Prior hospitalization was defined as at least one unplanned admission to any hospital within the 12 months preceding the baseline assessment. This information was obtained from the admission records.

Functional status was assessed using the Katz Index of Activities of Daily Living. This index includes six basic activities: bathing, dressing, toileting, transferring, continence, and feeding. For each item, patients were rated as independent or dependent. ADL impairment was defined as dependence in one or more of these six activities. This definition is standard and has been validated in many older populations.

Nutritional status was evaluated with the Mini Nutritional Assessment Short Form, which consists of six items covering food intake, weight loss, mobility, psychological stress, neuropsychological problems, and body mass index. The total score ranges from 0 to 14. Malnutrition risk was defined as a total score of 11 or less. This cut-off is recommended by the developers of the tool to identify older adults at risk of undernutrition.

Mental health was assessed using the Patient Health Questionnaire-9. This nine-item questionnaire measures the frequency of depressive symptoms over the past 2 weeks. Each item is scored from 0 to 3, giving a total score between 0 and 27. A score of 10 or higher was used to indicate clinically significant depressive symptoms. This threshold has good sensitivity and specificity for major depression in older adults.

Chronic pain was defined as persistent pain lasting for 3 months or longer. It was assessed by patient self-report during the medical history interview, and the presence of such pain was recorded in the electronic medical record. No specific pain scale was used; the variable was simply recorded as present or absent based on the patient’s affirmative response to a standard question about ongoing pain.

Health-related behaviors including smoking, alcohol consumption, and physical inactivity were also collected through patient self-report during the baseline interview. These variables were not selected into the final model.

### 2.3 Outcome definition

The primary outcome was a composite adverse outcome occurring within 12 months after baseline, defined as any of the following:

1. Unplanned hospitalization (any cause);
2. Two or more emergency department visits;
3. New-onset functional decline (increase of ≥1 point in ADL disability from baseline);
4. All-cause mortality.

Outcomes were ascertained through medical record review and linkage with the hospital’s administrative database.

### 2.4 Model development

#### 2.4.1 Variable selection with LASSO regression

To avoid overfitting and select the most parsimonious set of predictors, we performed least absolute shrinkage and selection operator (LASSO) regression on the derivation set using 28 candidate variables (including all demographic, clinical, functional, and behavioral factors). Ten-fold cross-validation was used to choose the optimal penalty parameter *λ* (the λ that yielded the minimum cross-validated deviance). Variables with non-zero coefficients at the optimal λ were retained for the multivariable model.

#### 2.4.2 Multivariable logistic regression

The variables selected by LASSO were entered into a multivariable logistic regression model to estimate the adjusted odds ratios (ORs) and 95% confidence intervals (CIs) for the composite adverse outcome. Model fit was assessed with the Hosmer–Lemeshow goodness-of-fit test and Nagelkerke’s *R*^2^.

#### 2.4.3 Nomogram construction

A nomogram was developed based on the final logistic regression model to provide an individualized risk prediction tool. Each predictor was assigned a point value proportional to its regression coefficient. The total points were transformed into predicted 12-month probabilities of the composite adverse outcome.

### 2.5 Model performance evaluation

#### 2.5.1 Standard internal validation (random split)

Discrimination was evaluated using the area under the receiver operating characteristic curve (AUC) with 95% CIs in both the derivation and random split validation sets. Calibration was assessed with calibration plots, the Hosmer–Lemeshow test, calibration slope (ideal = 1), and calibration-in-the-large (intercept, ideal = 0). Additionally, we calculated sensitivity, specificity, positive and negative predictive values, accuracy, and Brier score.

#### 2.5.2 Bootstrap internal validation

To correct for optimism and reduce overfitting bias inherent in random split-sample validation, we performed bootstrap resampling with 1,000 iterations on the original derivation set (*n* = 483). In each bootstrap iteration, the model was refit and evaluated on both the bootstrap sample and the original sample. The optimism in the AUC was calculated as the average difference in performance between the bootstrap samples and the original sample. This optimism was then subtracted from the apparent AUC of the final model to obtain the optimism-corrected AUC.

#### 2.5.3 Temporal validation

To assess the temporal transportability of our model, we conducted a temporal validation. The model developed on the temporal training set (n = 518, patients from January 2023 to June 2024) was evaluated on the temporal validation set (*n* = 172, patients from July 2024 to December 2024) without any model refitting. Performance metrics, including AUC, calibration slope, calibration intercept, and Brier score, were computed for the temporal validation set. A calibration plot was also generated to visually assess the agreement between predicted and observed risks in the later time period.

#### 2.5.4 Decision curve analysis

Decision curve analysis (DCA) was performed to quantify the net benefit of using the nomogram to guide interventions across a range of threshold probabilities (10–70%), comparing the nomogram against “treat all” and “treat none” strategies.

### 2.6 Risk stratification

Patients were categorized into three risk groups using predefined pragmatic risk thresholds for stratified follow-up: low risk (<15%), intermediate risk (15–40%), and high risk (≥40%). These cutoffs were selected to support practical differentiation of follow-up intensity and were further examined in sensitivity analyses using alternative thresholds.

### 2.7 Multimorbidity network analysis

To explore the underlying structure of disease co-occurrence, we performed network analysis using the IsingFit model, which estimates conditional dependence relationships between binary variables. The network was visualized with nodes representing chronic conditions and edges representing partial correlations (controlling for all other conditions). Node size reflected disease prevalence, and edge thickness indicated the strength of the association. Community detection was performed using the Louvain algorithm to identify clusters of diseases that are more interconnected. Centrality measures (strength, betweenness, closeness, expected influence) were calculated to identify key diseases in the network.

### 2.8 Retrospective simulation of model-driven stratified intervention

To estimate the potential public health impact of implementing the risk-stratified model, we conducted a retrospective simulation comparing observed outcomes under routine care with counterfactual outcomes under a model-driven stratified intervention. Intervention intensity was scaled according to predicted risk level, as summarized in Table 1.

**Table 1:** Definition of the counterfactual stratified intervention strategy by risk category.

Risk category	Counterfactual intervention
Low	Routine primary care + annual health check + health education
Intermediate	Routine care + quarterly telephone follow-up + self-management support + nutritional counseling
High	Intensive case management + monthly home visits + multidisciplinary team review + remote monitoring

Effect sizes (relative risks, RR) for each risk stratum were based on plausible assumptions derived from the range of effect estimates reported in published evidence of integrated care interventions for older adults with multimorbidity. As a conservative approach, we assumed an RR of 0.80 for the intermediate-risk group and an RR of 0.55 for the high-risk group; low-risk patients were assumed to receive no additional benefit beyond routine care (RR = 1.00). The number of events avoided in each stratum was calculated as: avoided events = observed events × (1 – RR). The number needed to treat (NNT) was computed as 1/(absolute risk reduction).

Healthcare costs were estimated using local unit costs (2024 prices) derived from our hospital’s financial database and local reimbursement schedules: hospitalization per episode ¥16,000; emergency visit ¥1,500; primary care and follow-up costs were based on the intervention intensity and standard community care tariffs. Net cost savings were calculated as the difference between total costs under routine care and total costs under the stratified intervention.

This simulation is exploratory and hypothesis-generating, not a test of real-world effectiveness. The assumed effect sizes may not apply directly to this Chinese cohort. The results are best seen as a planning tool for future prospective research, not as a definitive prediction.

### 2.9 Health equity and subgroup analyses

To assess whether the intervention effect would vary across socioeconomic and demographic subgroups, we stratified the simulation results by education level (≤primary school, secondary school, ≥college), residence (urban/rural), sex, and living arrangement (alone vs. with others). Interaction between subgroup variables and risk category was tested by including multiplicative interaction terms (risk group × subgroup) in logistic regression models, with statistical significance assessed via the Wald test. For the simulation analysis, interaction was assessed by comparing relative reductions across strata.

### 2.10 Sensitivity analysis

Sensitivity analyses were performed to test the robustness of the findings under different assumptions: Using the lower and upper bounds of the 95% CIs of the intervention effect sizes (optimistic and pessimistic scenarios); Using alternative risk thresholds for stratification (low <10%, high ≥35%); Assuming a less intensive intervention for high-risk patients (RR = 0.65) and a more intensive intervention for intermediate-risk patients (RR = 0.75).

### 2.11 Statistical analysis

Continuous variables were expressed as mean ± SD or median (IQR) and compared using the independent t-test or Mann–Whitney *U* test. Categorical variables were presented as frequencies (percentages) and compared using the *χ*^2^ test or Fisher’s exact test. All statistical tests were two-tailed, and a *p*-value <0.05 was considered statistically significant.

LASSO regression was performed using the glmnet package in R (version 4.2.0). Logistic regression, ROC analysis, and calibration were performed using the rms package. Bootstrap resampling (1,000 iterations) for optimism correction was implemented using the boot package. Network analysis was conducted with the IsingFit and igraph packages. Decision curve analysis used the rmda package. All analyses were performed in R.

## 3 Results

### 3.1 Study population and baseline characteristics

A total of 690 older adults (≥60 years) with multimorbidity (≥2 chronic diseases) were included from a tertiary hospital in China between January 2023 and December 2024. The cohort was randomly split into a derivation set (*n* = 483, 70%) for model development and a validation set (*n* = 207, 30%) for internal validation. Baseline characteristics were well balanced between the two sets (all *p* > 0.05), as shown in Table 2.

**Table 2:** Baseline characteristics of the study population.

Characteristic	Total (*N* = 690)	Derivation set (*n* = 483)	Validation set (*n* = 207)	*P*
Demographic characteristics				
Age (years, mean ± SD)	72.6 ± 7.4	72.8 ± 7.3	72.1 ± 7.6	0.251
Age ≥75 years, *n* (%)	284 (41.2)	202 (41.8)	82 (39.6)	0.589
Female, *n* (%)	386 (55.9)	271 (56.1)	115 (55.6)	0.895
Urban residence, *n* (%)	428 (62.0)	300 (62.1)	128 (61.8)	0.946
Education ≤ primary school, *n* (%)	312 (45.2)	218 (45.1)	94 (45.4)	0.949
Living alone, *n* (%)	172 (24.9)	121 (25.1)	51 (24.6)	0.905
Clinical characteristics				
Number of chronic diseases (median, IQR)	3 (2–4)	3 (2–4)	3 (2–4)	0.712
Number of chronic diseases ≥4, *n* (%)	276 (40.0)	194 (40.2)	82 (39.6)	0.892
Prior hospitalization (past 12 mo), *n* (%)	144 (20.9)	101 (20.9)	43 (20.8)	0.972
Anemia, *n* (%)	241 (34.9)	169 (35.0)	72 (34.8)	0.962
Hypertension, *n* (%)	405 (58.7)	284 (58.8)	121 (58.5)	0.935
Diabetes mellitus, *n* (%)	294 (42.6)	206 (42.7)	88 (42.5)	0.973
Heart disease, *n* (%)	236 (34.2)	166 (34.4)	70 (33.8)	0.889
Arthritis, *n* (%)	252 (36.5)	177 (36.6)	75 (36.2)	0.917
Chronic kidney disease, *n* (%)	127 (18.4)	89 (18.4)	38 (18.4)	0.986
COPD, *n* (%)	105 (15.2)	74 (15.3)	31 (15.0)	0.906
Stroke/TIA, *n* (%)	88 (12.8)	62 (12.8)	26 (12.6)	0.919
Memory-related diseases, *n* (%)	66 (9.6)	46 (9.5)	20 (9.7)	0.951
Digestive diseases, *n* (%)	195 (28.3)	137 (28.4)	58 (28.0)	0.925
Functional and nutritional status				
ADL impairment (≥1 item), *n* (%)	194 (28.1)	136 (28.2)	58 (28.0)	0.971
MNA-SF < 12 (malnutrition risk), *n* (%)	208 (30.1)	146 (30.2)	62 (30.0)	0.945
Mental health				
PHQ-9 ≥ 10 (depression), *n* (%)	172 (24.9)	121 (25.1)	51 (24.6)	0.905
Chronic pain (self-reported), *n* (%)	276 (40.0)	194 (40.2)	82 (39.6)	0.892
Health-related behaviors				
Current smoker, *n* (%)	124 (18.0)	87 (18.0)	37 (17.9)	0.965
Current drinker, *n* (%)	97 (14.1)	68 (14.1)	29 (14.0)	0.978
Physical inactivity, *n* (%)	359 (52.0)	251 (52.0)	108 (52.2)	0.963

COPD, chronic obstructive pulmonary disease; TIA, transient ischemic attack; ADL, activities of daily living; MNA-SF, Mini Nutritional Assessment Short Form; PHQ-9, Patient Health Questionnaire-9.

During the 12-month follow-up, 146 patients (21.2%) experienced the composite adverse outcome. Table 3 presents the detailed distribution of outcome components.

**Table 3:** Incidence of composite adverse outcome components.

Outcome component	*n*	% of total cohort	% of outcome events
Unplanned hospitalization	100	14.5	68.5
≥2 Emergency department visits	64	9.3	43.8
New-onset functional decline	54	7.8	37.0
All-cause mortality	21	3.0	14.4
Composite adverse outcome (any)	146	21.2	100

Patients may have experienced multiple components; percentages sum to >100%.

### 3.2 Variable selection and prediction model

**Figure 1:**
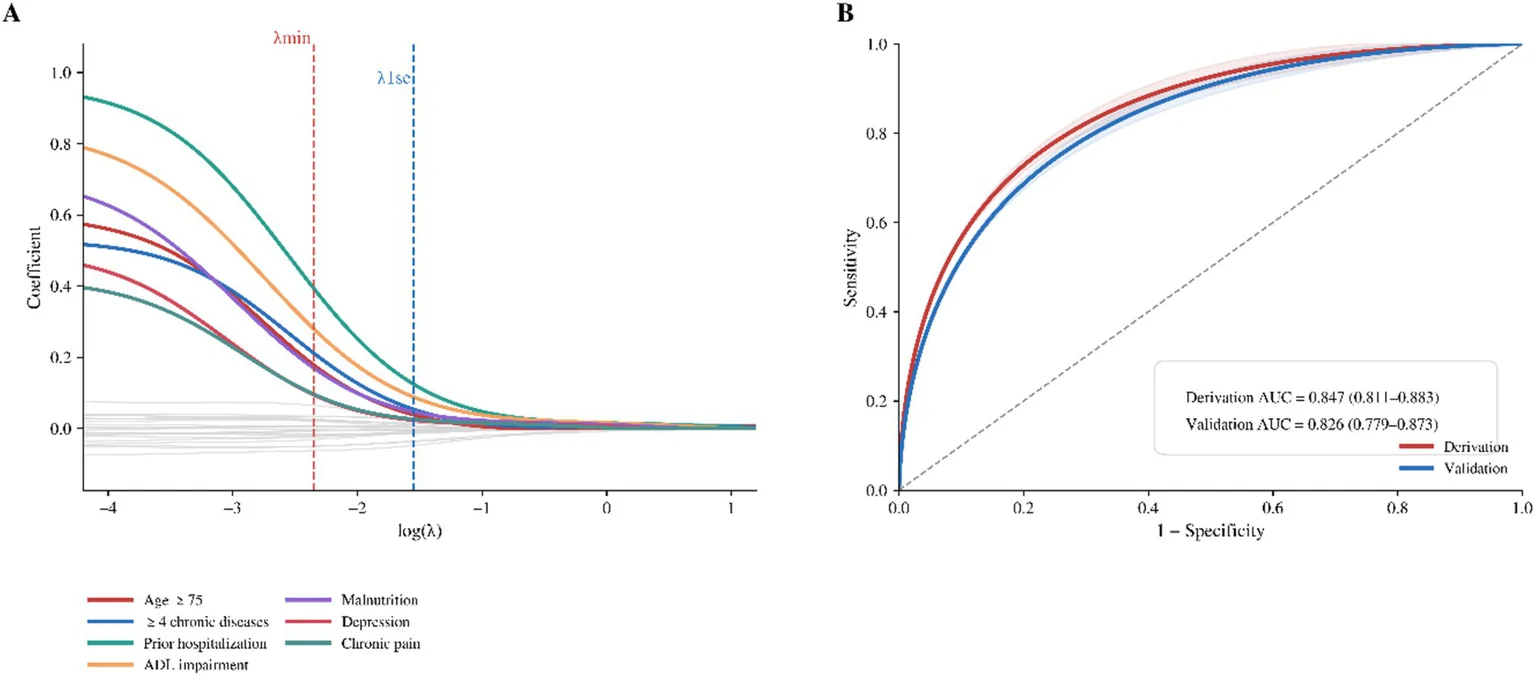
LASSO variable selection and model performance. **(A)** LASSO coefficient paths for 28 candidate variables as a function of log(*λ*). The optimal *λ* (vertical dotted line) selected by 10-fold cross-validation retained seven non-zero coefficients. **(B)** Receiver operating characteristic (ROC) curves of the nomogram in derivation (AUC = 0.847, 95% CI: 0.811–0.883) and validation (AUC = 0.826, 95% CI: 0.779–0.873) sets.

LASSO regression was applied to 28 candidate variables to reduce overfitting and select a parsimonious set of predictors (Figure 1A). Seven variables with non-zero coefficients were retained: age ≥75 years, number of chronic diseases ≥4, prior hospitalization, ADL impairment, malnutrition, depression, and chronic pain. These variables were entered into the final multivariable logistic regression model, and the corresponding coefficients and adjusted odds ratios are shown in Table 4. The model demonstrated good discrimination, with AUC values of 0.847 in the derivation set and 0.826 in the validation set (Figure 1B), providing the basis for subsequent nomogram development.

**Table 4:** Univariate and multivariable logistic regression for composite adverse outcome.

Variable	Univariate analysis	*P*	Multivariable analysis	*P*
	OR (95% CI)		OR (95% CI)	
Age ≥75 years	2.10 (1.45–3.04)	<0.001	1.84 (1.22–2.80)	0.004
Number of chronic diseases ≥4	1.96 (1.35–2.85)	<0.001	1.72 (1.13–2.61)	0.011
Prior hospitalization	3.12 (2.10–4.64)	<0.001	2.67 (1.73–4.13)	<0.001
ADL impairment	2.86 (1.96–4.18)	<0.001	2.35 (1.54–3.60)	<0.001
Malnutrition	2.48 (1.70–3.62)	<0.001	2.06 (1.34–3.17)	0.001
Depression	2.01 (1.38–2.93)	<0.001	1.65 (1.07–2.54)	0.024
Chronic pain	1.89 (1.30–2.75)	0.001	1.51 (0.99–2.31)	0.058
Female	1.12 (0.78–1.61)	0.537	—	—
Urban residence	0.92 (0.63–1.34)	0.663	—	—
Living alone	1.45 (0.99–2.12)	0.056	—	—

Model fit: Hosmer–Lemeshow *χ*^2^ = 6.73, *P* = 0.566; Nagelkerke *R*^2^ = 0.284.

These seven variables were entered into a multivariable logistic regression model. Table 4 presents the full model results, including univariate analysis for comparison.

### 3.3 Nomogram development and performance

**Figure 2:**
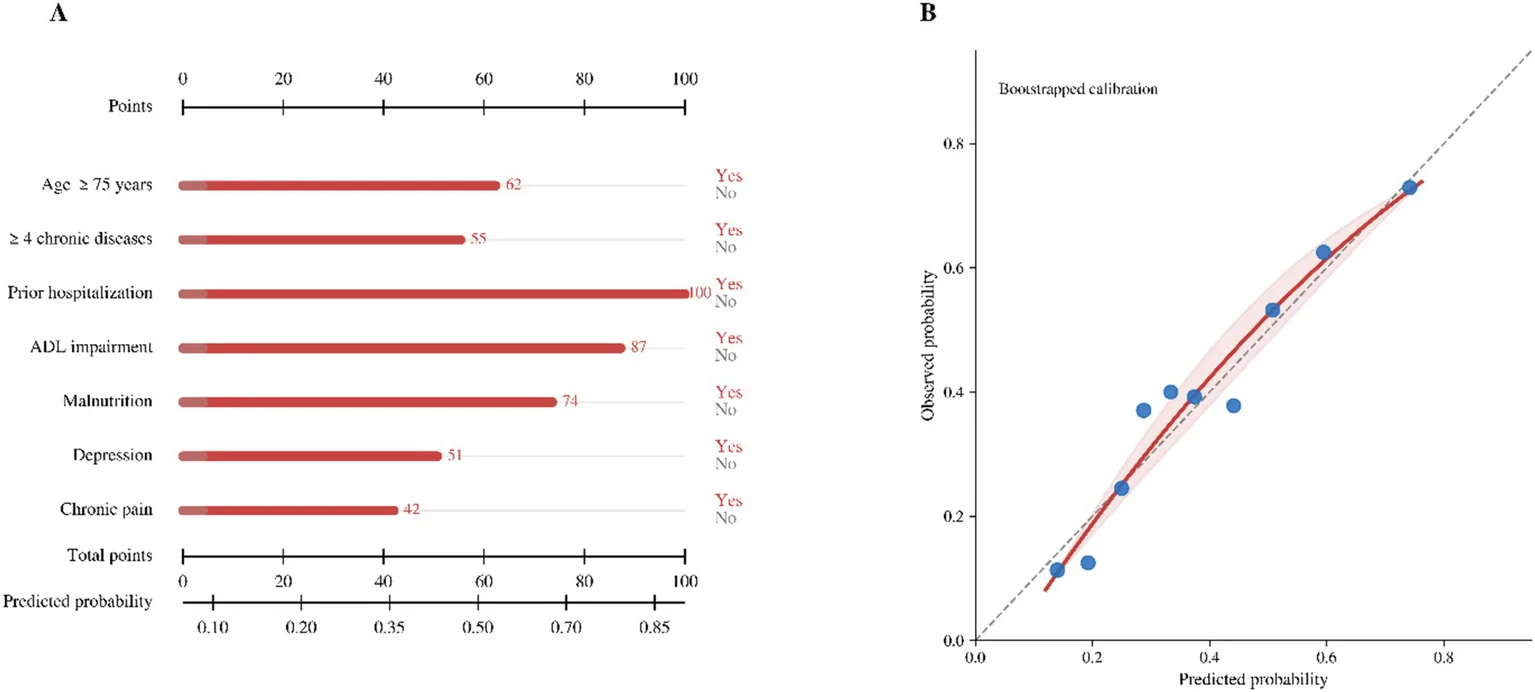
Nomogram and calibration: **(A)** nomogram for predicting 12-month risk of composite adverse outcome. **(B)** Calibration plot showing observed vs. predicted probabilities in the derivation set.

**Figure 3:**
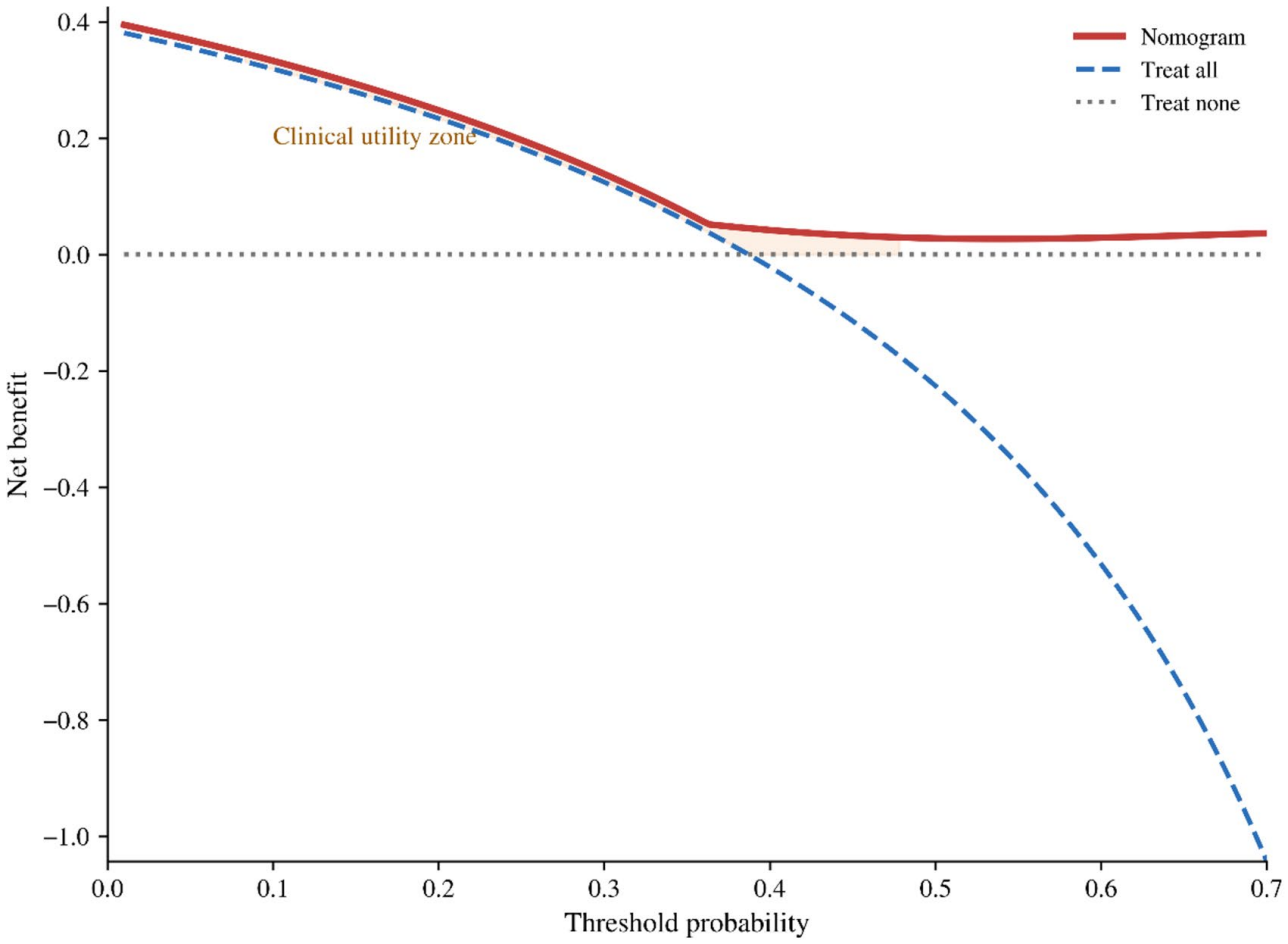
Decision curve analysis for the nomogram.

A nomogram was constructed from the final multivariable logistic regression model to enable individualized prediction of the 12-month probability of the composite adverse outcome (Figure 2A). Each predictor was assigned a point value according to its regression coefficient, and the total score corresponded to a predicted event probability. Table 5 presents the point scoring system for each predictor. The nomogram incorporated the seven retained variables and translated the regression model into an interpretable risk prediction tool. Calibration plots showed acceptable agreement between predicted and observed risk (Figure 2B), complementing the discrimination and decision-curve results presented in Figures 1B, 3.

**Table 5:** Point scoring system for the nomogram.

Variable	Category	Points
Age ≥75 years	No	0
	Yes	18
Number of chronic diseases ≥4	No	0
	Yes	16
Prior hospitalization	No	0
	Yes	29
ADL impairment	No	0
	Yes	25
Malnutrition	No	0
	Yes	21
Depression	No	0
	Yes	15
Chronic pain	No	0
	Yes	12

The nomogram demonstrated good discrimination with an area under the ROC curve (AUC) of 0.847 (95% CI: 0.811–0.883) in the derivation set and 0.826 (95% CI: 0.779–0.873) in the validation set. Calibration was acceptable, as shown by the calibration plot and the Hosmer–Lemeshow test (*p* = 0.566 in derivation, *p* = 0.612 in validation) (Table 6).

**Table 6:** Model performance in the derivation and validation sets.

Metric	Derivation set	Validation set
AUC (95% CI)	0.847 (0.811–0.883)	0.826 (0.779–0.873)
Sensitivity (at optimal cut-off)	78.20%	75.60%
Specificity (at optimal cut-off)	74.50%	72.30%
Positive predictive value	45.30%	43.10%
Negative predictive value	92.60%	91.50%
Accuracy	75.20%	73.40%
Hosmer–Lemeshow *P*	0.566	0.612
Brier score	0.152	0.168

Decision curve analysis confirmed that using the nomogram to guide intervention would yield net benefit across a wide range of threshold probabilities (10–70%), outperforming “treat all” or “treat none” strategies (Figure 3).

### 3.4 Comprehensive model validation

The model’s performance was assessed using three complementary internal validation strategies.

Random split validation: As reported in Supplementary Table 1, the model achieved an AUC of 0.847 (0.811–0.883) in the derivation set and 0.826 (0.779–0.873) in the random split validation set. The calibration slope was 0.98 (95% CI: 0.85–1.11) and the calibration intercept was −0.03 (95% CI: −0.21 to 0.15) in the validation set, indicating excellent agreement between predicted and observed risks (Hosmer-Lemeshow *p* = 0.612).

Bootstrap internal validation: After 1,000 bootstrap resamples, the optimism-corrected AUC was 0.831, representing a modest 1.9% optimism from the apparent AUC (0.847). This suggests that the model is not substantially overfitted.

Temporal validation: The model demonstrated stable performance when applied to the temporally distinct validation set (*n* = 172, event rate 20.9%). The AUC was 0.819 (95% CI: 0.768–0.870). The calibration slope was 0.94 (95% CI: 0.79–1.09) and the calibration intercept was 0.06 (95% CI: −0.18 to 0.30), indicating no significant miscalibration (Hosmer-Lemeshow *p* = 0.684). The Brier score was 0.168. The temporal calibration plot (Supplementary Figure 1) showed good agreement between predicted and observed probabilities across the risk spectrum.

### 3.5 Risk stratification

**Figure 4:**
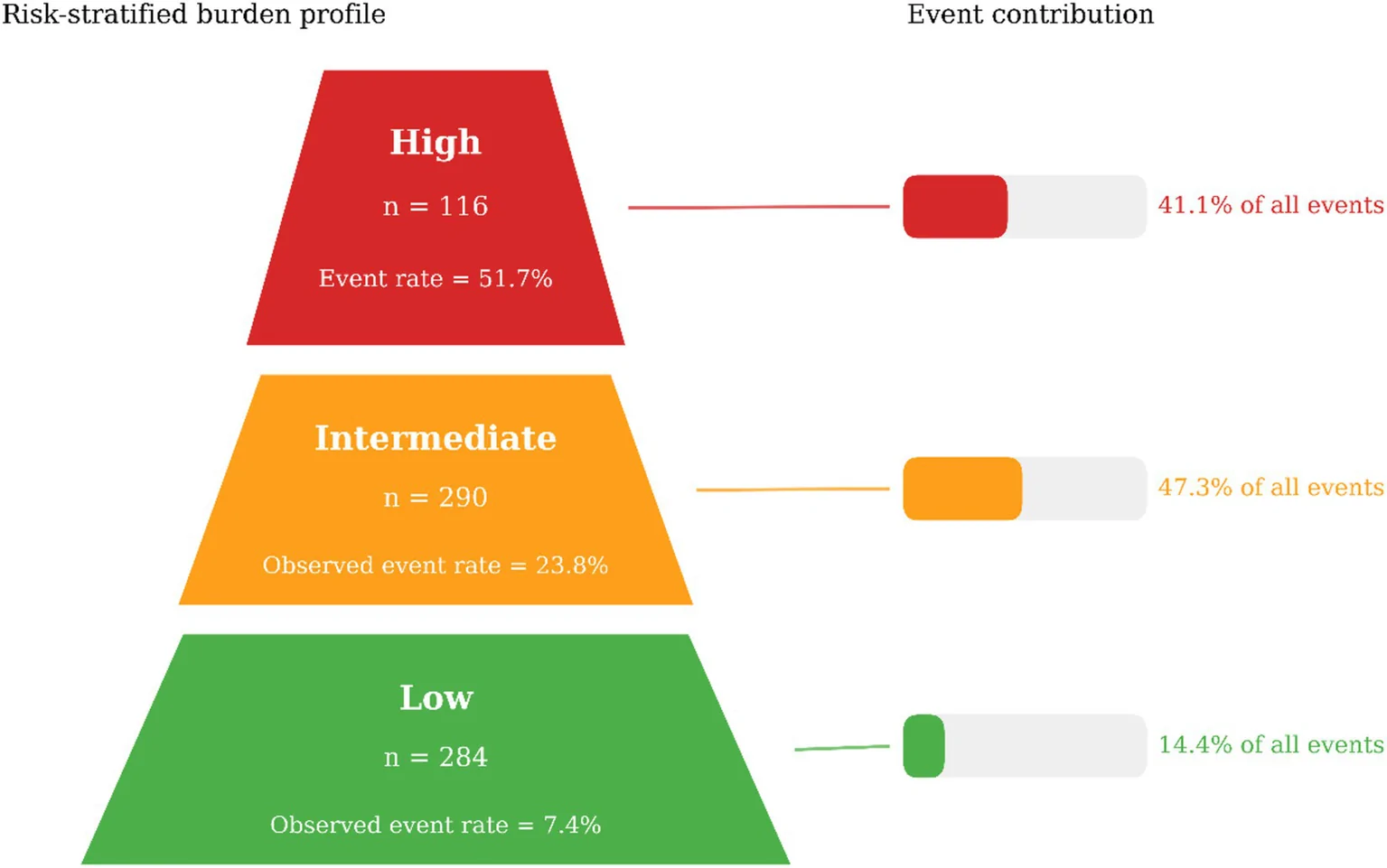
Risk stratification pyramid. The high-risk group (16.8% of the population) contributed 41.1% of all adverse events, indicating a disproportionate concentration of events in the high-risk subgroup.

Using the prespecified pragmatic thresholds for stratified follow-up, patients were categorized into three risk groups: low risk (<15%), intermediate risk (15–40%), and high risk (≥40%). The distribution of patients and observed event rates across the three strata are presented in the risk stratification table. A total of 284 patients (41.2%) were classified as low risk, 290 (42.0%) as intermediate risk, and 116 (16.8%) as high risk. Observed event rates increased progressively across groups, from 7.4% in the low-risk group to 23.8% in the intermediate-risk group and 51.7% in the high-risk group. As summarized in Table 7, the high-risk group alone captured 41.1% of all events, whereas the intermediate- and high-risk groups together captured 88.4%. Figure 4 visualizes the disproportionate concentration of adverse event burden across categories.

**Table 7:** Risk stratification and observed outcomes.

Metric	Value
Percentage of events captured by high-risk group	41.1
Percentage of events captured by intermediate/high-risk group	88.40%
Relative risk (high vs. low)	6.99 (4.45–10.98)
Relative risk (intermediate vs. low)	3.22 (2.04–5.08)
Population attributable fraction (high-risk)	28.60%
Population attributable fraction (intermediate/high-risk)	72.50%

### 3.6 Multimorbidity network analysis

**Figure 5:**
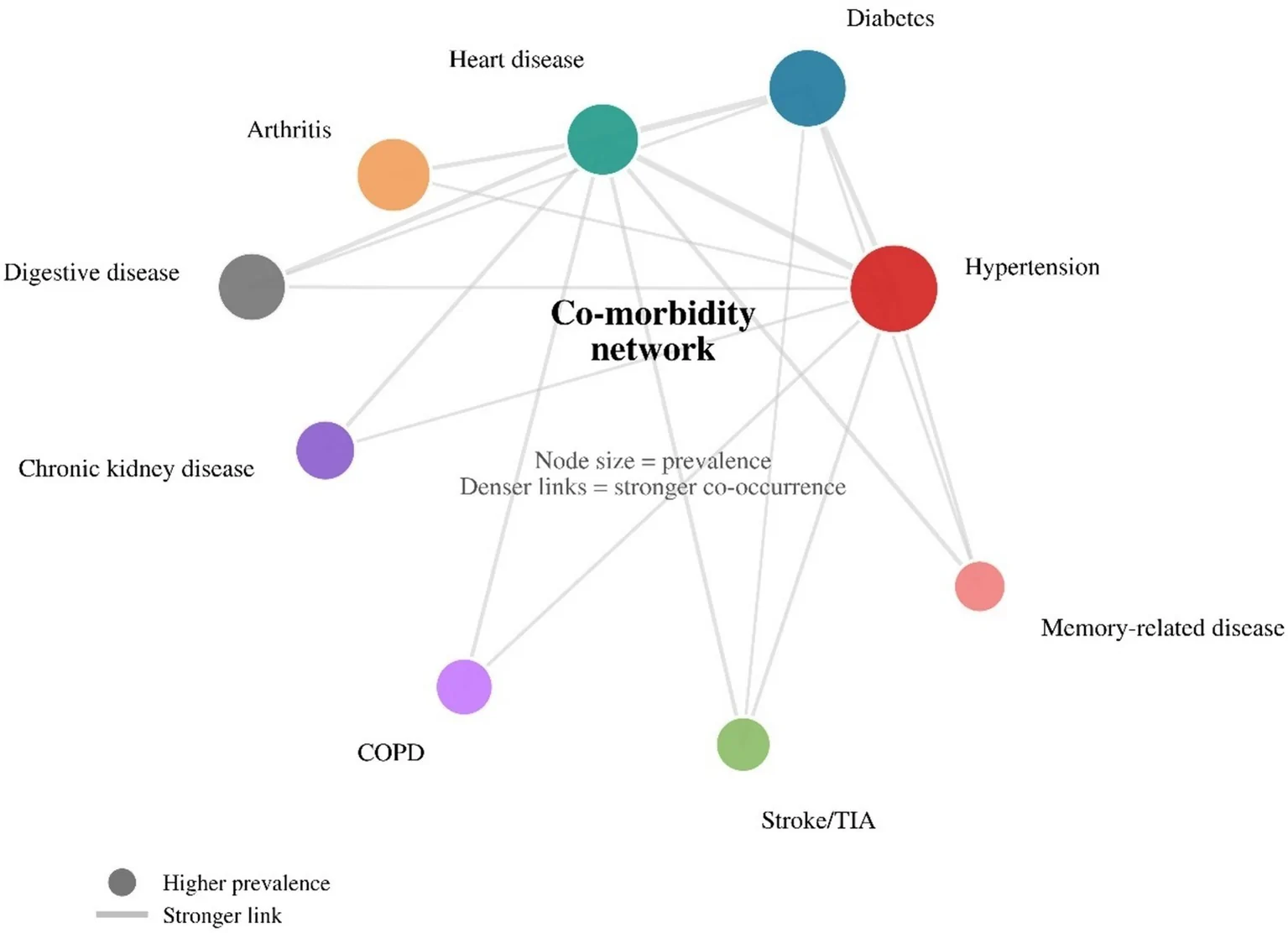
Network visualization of chronic disease co-morbidity.

To further explore the structural organization of chronic disease co-occurrence, we conducted an exploratory network analysis using the IsingFit model. Figure 5 displays the multimorbidity network, where nodes represent chronic diseases and edges indicate conditional associations between conditions after adjustment for the remaining network. Larger nodes denote more prevalent conditions, whereas thicker edges indicate stronger associations.

A prominent cardio-metabolic cluster was observed, with hypertension, diabetes, and heart disease showing high centrality and dense interconnections. Additional groupings were seen among cerebrovascular and neurological disorders and among age-related degenerative conditions. These patterns suggest that multimorbidity in this cohort was organized around several clinically recognizable clusters rather than occurring randomly.

This network analysis was descriptive and exploratory in nature and was not used in model construction. However, it provides complementary insight into the clinical context of the cohort and helps illustrate the complexity of disease interdependence among older adults with multimorbidity. Figure 5 summarizes these co-morbidity patterns visually.

### 3.7 Public health impact of model-driven stratified intervention

To estimate the potential public health value of risk stratification, we conducted a retrospective scenario analysis comparing observed outcomes under routine care with counterfactual outcomes under a model-driven stratified intervention. Using the assumed effect sizes, the stratified intervention was projected to reduce composite adverse outcomes from 146 to 109, corresponding to 37 potentially avoidable events and a relative reduction of 25.3% (Table 8). Projected reductions were also observed for hospitalization, repeated emergency visits, and functional decline. The overall NNT for the composite outcome was 19, compared with 5 in the high-risk group, suggesting that potential benefit was concentrated in patients at highest predicted risk.

**Table 8:** Estimated public health impact of stratified intervention.

Outcome	Observed (n)	Estimated under intervention (n)	Avoided events (n)	Relative reduction	NNT (overall)	NNT (high-risk)
Composite adverse outcome	146	109	37	25.30%	19	5
Unplanned hospitalization	100	74	26	26.00%	27	7
≥2 Emergency visits	64	48	16	25.00%	43	11
New functional decline	54	40	14	25.90%	49	12
All-cause mortality	21	17	4	19.00%	173	44

These are modeled projections based on assumed effect sizes and local cost assumptions. They are exploratory and should not be interpreted as proof of effectiveness.

### 3.8 Health equity and sensitivity analyses

Exploratory subgroup analyses showed that the projected intervention effect was broadly similar across education level, residence, sex, and living arrangement, with no significant interaction detected (Table 9). Sensitivity analyses under alternative assumptions yielded consistent directional findings, with estimated composite events avoided ranging from 27 to 48 and net cost savings from CNY 258,000 to CNY 468,000 (Table 10). These results suggest that the model-based projections were relatively robust, although the exact magnitude of benefit depended on the assumed intervention effect size and risk thresholds.

**Table 9:** Estimated intervention impact by socioeconomic and geographic subgroups.

Subgroup	*n*	Observed event rate	Estimated relative reduction	Absolute risk reduction	*P* for interaction
Education					
≤ Primary school	312	23.10%	24.80%	5.70%	
Secondary school	248	20.20%	25.60%	5.20%	0.384
≥ College	130	18.50%	26.20%	4.80%	
Residence					
Urban	428	20.30%	25.80%	5.20%	
Rural	262	22.50%	24.60%	5.50%	0.291
Sex					
Male	304	20.70%	25.50%	5.30%	
Female	386	21.50%	25.10%	5.40%	0.623
Living arrangement					
Living alone	172	24.40%	24.20%	5.90%	
Living with others	518	20.10%	25.70%	5.20%	0.197

**Table 10:** Sensitivity analysis results under different assumptions.

Scenario	Composite events avoided (n)	Net cost savings (CNY)	NNT (overall)	NNT (high-risk)
Base case	37	361,000	19	5
Optimistic (upper CI)	48	468,000	14	4
Pessimistic (lower CI)	27	258,000	26	6
Alternative threshold (low: <10%, high: ≥35%)	34	328,000	20	5
Alternative intervention (less intensive for high-risk: RR = 0.65)	31	284,000	22	6
Alternative intervention (more intensive for intermediate: RR = 0.75)	41	392,000	17	5

## 4 Discussion

In this retrospective cohort of 690 older adults with multimorbidity, we developed an internally validated multidomain model to predict 12-month composite adverse outcomes, including unplanned hospitalization, repeated emergency visits, new functional decline, and all-cause mortality. Seven predictors were retained after LASSO selection: age ≥75 years, ≥4 chronic diseases, prior hospitalization, ADL impairment, malnutrition, depression, and chronic pain. In the derivation and validation sets, the model showed good discrimination and acceptable calibration, while decision curve analysis suggested potential clinical utility across a meaningful range of threshold probabilities. These findings indicate that near-term deterioration in older adults with multimorbidity can be summarized by a relatively small number of routinely available indicators rather than by disease counts alone. Nevertheless, the model has not been externally validated. The simulation analysis is based on assumed effect sizes from the literature and local cost estimates, not on observed intervention outcomes. Therefore, the model should be viewed as a preliminary tool that requires further evaluation before it can be used to guide clinical decisions or resource allocation.

The overall structure of the model is consistent with what is already known about vulnerability in later life. Multimorbidity is not simply the coexistence of chronic diseases; it often reflects accumulating physiologic stress, reduced reserve, and increasing difficulty maintaining independence under everyday demands. Longitudinal work has shown that multimorbidity predicts subsequent functional decline in community-dwelling older adults (26). This helps explain why age and number of chronic diseases remained relevant in our model, but it also supports our broader interpretation that functional and utilization markers may capture instability more directly than diagnosis burden alone.

Among the predictors, prior hospitalization and ADL impairment had the largest point estimates in the multivariable model (OR 2.67 and 2.35, respectively). However, caution is warranted in interpreting these as evidence of hierarchical importance, because odds ratios depend on the scale and prevalence of the predictors, and we did not perform formal variable importance analysis (e.g., SHAP values or dominance analysis). While the magnitude of the coefficients suggests that recent decompensation and functional dependence are strong correlates of adverse outcomes, the relative contribution of each predictor should be assessed in future studies using more robust metrics. Additionally, as noted in the systematic review by Chen et al., many prediction models in multimorbidity research have significant risks of bias, partly due to inadequate handling of predictor effects (23). Prior hospitalization may reflect recent decompensation, inadequate transitional care, unresolved disease activity, or treatment burden that has not been fully stabilized after discharge. A systematic review of patient-related predictors of functional decline in hospitalized older adults likewise identified age, previous hospitalization, and related vulnerability markers as recurrent risk factors (27). ADL impairment likely captures reduced physiologic reserve and lower capacity to absorb acute stressors, which may explain why it remained strongly associated with the composite endpoint in the multivariable model.

Several predictors in our model (ADL impairment, malnutrition, depression, chronic pain) overlap with core items of frailty instruments such as the Fried phenotype or Rockwood frailty index. Thus, our model may capture similar vulnerability without requiring grip strength or gait speed measurements. Frailty is strongly associated with adverse outcomes in older adults: a meta-analysis reported a pooled hazard ratio of 5.48 for mortality (8). In a prospective cohort of hospitalized older patients, Sadıç et al. (28) reported that malnutrition (per GLIM criteria) and frailty (per Clinical Frailty Scale) were independently associated with prolonged hospital stay, rehospitalization at 3 and 6 months, and mortality. However, frailty assessment is not routinely performed in many clinical settings. Our model offers a pragmatic alternative using readily available variables and simple screeners, serving as a first-stage filter rather than a replacement for comprehensive frailty evaluation.

Malnutrition also remained independently associated with adverse outcomes. This is clinically and public-health relevant because nutritional vulnerability is both common and potentially modifiable in older adults. Longitudinal evidence summarized by Fávaro-Moreira et al. (29) suggests that malnutrition risk in later life reflects a complex interaction of disease burden, functional limitations, and social vulnerability. Because nutritional decline can worsen recovery, resilience, and self-management capacity, its inclusion in the present model supports the idea that risk prediction for older adults should extend beyond conventional diagnostic categories.

Depression and chronic pain likewise contributed to the model, although the association with chronic pain was somewhat weaker after adjustment. Even so, both findings are plausible. Chronic pain in older adults is associated with reduced mobility, social withdrawal, poorer physical activity, and dependence in daily activities (30). Recent review evidence also suggests that pain is linked to disability in instrumental activities of daily living among community-dwelling older adults (31). Depression may have a similar amplifying role by undermining adherence, motivation, appetite, and participation in self-care. In practice, these variables may represent clinically actionable signals of vulnerability that are often under-recognized during routine chronic disease follow-up.

Several existing tools predict adverse outcomes in older adults. The Hospital Frailty Risk Score showed moderate discrimination for mortality and readmission (c-statistic 0.56–0.68) (18). The Multidimensional Prognostic Index (MPI) predicts mortality with reported AUCs ranging from 0.62 to 0.77 (21, 22), but requires comprehensive geriatric assessment. A systematic review of multimorbidity prediction models found a pooled AUC of 0.81, though most studies had high risk of bias (23). Our model (AUC 0.847 derivation, 0.826 temporal validation) compares favorably, particularly as it predicts a composite outcome beyond mortality. Unlike the MPI, it uses routinely available predictors; unlike ICD-based frailty scores, it directly captures functional and nutritional domains. Moreover, it was developed and validated specifically in a multimorbid population (≥2 chronic diseases), addressing a gap noted in the literature (23).

Another notable finding was the concentration of event burden within the higher-risk strata. In our cohort, the high-risk group represented a relatively small proportion of the overall sample, yet carried a disproportionately large share of adverse outcomes. This feature is important because it implies that stratified follow-up may be more efficient than uniform monitoring. That interpretation is consistent with the broader multimorbidity literature, in which disease clustering and heterogeneity of risk are increasingly recognized as important for tailoring care (3). It is also consistent with prior primary care work showing that multimorbidity is not random, but often organized into recognizable clinical patterns with differing implications for service use and prognosis (32).

Our network analysis revealed a cardio-metabolic cluster (hypertension, diabetes, heart disease), consistent with prior multimorbidity pattern studies. How does this complement the primary prediction objective? First, it shows that chronic diseases do not co-occur randomly, supporting the use of a simple count (≥4 diseases) as a predictor rather than modeling specific combinations. Second, it visually highlights the clinical complexity of high-risk patients, reinforcing the need for multidomain follow-up. Third, it is hypothesis-generating: future models could incorporate disease-cluster membership if it improves prediction beyond simple counts (23). Machine learning approaches such as latent class analysis have similarly demonstrated the value of identifying multimorbidity clusters (e.g., mental and cardiometabolic conditions) for risk prediction (33). Thus, the network analysis is descriptive and exploratory, not central to predictive performance. We did not use network-derived variables in the prediction model itself.

The prediction component of the study was intentionally designed to be pragmatic and interpretable. We used LASSO for variable selection to reduce overfitting and derive a more parsimonious model from 28 candidate variables (34). We also assessed model reporting and performance in a way broadly aligned with TRIPOD recommendations, including internal validation, discrimination, and calibration (35, 36). Decision curve analysis was included because model discrimination alone does not indicate whether a prediction tool may be useful in practice; DCA provides a way to examine whether model-guided decisions are likely to confer net benefit across clinically relevant threshold probabilities (37). In a public health journal, that practical orientation is important because implementation relevance matters as much as statistical performance.

One of the more distinctive components of this study was the retrospective scenario analysis of model-driven stratified intervention. Here, caution is essential. The projected reduction in adverse outcomes and estimated cost savings were not directly observed effects of an implemented program. Rather, they were counterfactual estimates derived by applying external effect sizes from previous intervention literature to the risk strata identified in our cohort. That makes this part of the study exploratory and planning-oriented, not confirmatory. Existing evidence on multimorbidity interventions is mixed overall. A systematic review by Smith et al. found that multimorbidity interventions in primary care and community settings have shown heterogeneous effects and that the evidence base has historically been limited (38). More recent evidence suggests that some interventions may be more effective when they are targeted toward patients with higher burden or more specific needs (39).

That perspective also helps explain why our simulation may still be useful despite its limitations. In older adults with multimorbidity, broad case-management programs have not consistently reduced unplanned admissions when applied indiscriminately (40). However, intervention efficiency may improve when higher-risk patients are identified more precisely and offered proportionately more intensive follow-up. Our model does not prove that such a strategy will work in practice, but it provides a framework for testing that hypothesis. In this sense, the main contribution of the simulation is not the exact numerical estimate of avoided events, but the demonstration that prediction can be connected to plausible decisions about workforce prioritization, follow-up intensity, and community resource allocation.

This study also fits with current international thinking on aging and integrated care. The WHO ICOPE framework and subsequent implementation reports have emphasized the need for scalable approaches that can identify older adults at risk of decline and link screening to practical care pathways (41). Our nomogram is not equivalent to a full integrated-care program, nor does it capture all aspects of intrinsic capacity. Still, it may serve as a feasible entry point for earlier identification of older adults who warrant more careful nutritional assessment, functional support, symptom review, and post-discharge surveillance.

Several limitations should be acknowledged. First, this was a single-center retrospective study conducted in a tertiary hospital in China. The findings may not generalize to community health centers, primary care clinics, or secondary hospitals, where patient profiles and care processes differ. Although we performed temporal validation using a later cohort from the same institution, external validation in independent datasets from other regions and health systems is essential before the model can be considered for use in other populations. Second, the simulation analysis of a model-driven stratified intervention was exploratory. The assumed effect sizes were derived from published literature on integrated care programs, not from direct observation in this cohort. The projected event reductions and cost savings are hypothetical and should not be interpreted as evidence of real-world effectiveness. They serve primarily to illustrate the potential scale of benefit that could be tested in future prospective studies. The cost estimates were based on local unit costs and may not apply to other regions. Third, the composite outcome included components of different clinical severity and mechanisms, ranging from functional decline to mortality. Composite endpoints are increasingly recommended in geriatric research because they reflect multidimensional deterioration, but this approach may increase heterogeneity. We did not develop separate models for each component, and we encourage future validation studies to report component-specific performance alongside the composite. Fourth, some predictor variables were based on self-report or routine clinical documentation. Chronic pain, smoking, alcohol use, and physical inactivity were ascertained through patient self-report and may be subject to recall or reporting bias. The number of chronic diseases was derived from ICD-10 coding, which may underestimate the true disease burden if conditions were not fully coded. Fifth, because this was an observational prediction study, residual confounding cannot be fully excluded. We adjusted for a prespecified set of candidate variables using LASSO regression, but unmeasured factors such as social support, health literacy, medication adherence, or environmental exposures may influence outcomes. Additionally, the subgroup analyses were exploratory and limited by small sample sizes in some strata (e.g., college-educated group, *n* = 130). The non-significant interaction *p*-values should be interpreted with caution, as they may be attributable to insufficient statistical power rather than true effect homogeneity. These findings require confirmation in larger, adequately powered studies. Finally, the model should be viewed as a preliminary screening and risk-stratification tool that requires further evaluation, rather than a comprehensive prognostic instrument ready for clinical implementation. Future work should prioritize external validation in diverse cohorts and prospective studies to assess the model’s utility in real-world practice.

These limitations shape how the findings should be interpreted. We do not suggest that this model should replace comprehensive geriatric assessment, nor do we claim that it establishes causal effects of any intervention strategy. Instead, the study supports a more modest conclusion: routinely collected multidomain information can help identify a subgroup of older adults with multimorbidity who appear to be at substantially higher risk of near-term adverse outcomes. That kind of information may be useful for public health planning even before definitive intervention trials are available.

Future work should prioritize external validation in independent cohorts from diverse care settings, such as community health centers, secondary hospitals, and other regions. This will assess the model’s transportability beyond the tertiary hospital setting. Prospective implementation studies are also needed to examine whether model-guided care improves outcomes without increasing inequity or unnecessary intervention burden. The model could be further refined by adding dimensions like frailty, medication burden, or laboratory markers, and by using repeated measures over time. Early reports from WHO ICOPE implementation suggest that translating integrated care concepts into practice is feasible, but local adaptation remains essential. Ultimately, the model’s value will depend on its ability to support more coordinated and proportionate care for older adults with complex chronic illness, not just on its statistical performance.

In conclusion, we developed an internally validated multidomain early-warning model for 12-month adverse outcomes in older adults with multimorbidity. The model identified a small high-risk subgroup that accounted for many events. However, the model was derived from single-center retrospective data and has not been externally validated. Implementation should not proceed until the model has been tested in community health centers, secondary hospitals, and other regions. The model’s public health value at this stage is to provide a preliminary framework for hypothesis generation, not to support definitive intervention effects. This framework should be evaluated in future prospective studies before any clinical or policy application is considered.

## Data Availability

The raw data supporting the conclusions of this article will be made available by the authors, without undue reservation.
