# Supplementary material for: A multidomain early warning model for 12-month adverse outcomes in older adults with multimorbidity: a risk-stratified public health approach
Source: Front Public Health. 2026 Sep 9;14:1841984. doi: 10.3389/fpubh.2026.1841984 (PMC13597775; doi:10.3389/fpubh.2026.1841984)
Supplement: Supplementary file 1 [file Data_sheet_1.docx]

**Supplementary Table 1. Summary of model performance across validation strategies**

| **Validation strategy** | **Sample size (n)** | **Event rate (%)** | **AUC (95% CI)** | **Calibration slope (95% CI)** | **Calibration-in-the-large (intercept, 95% CI)** | **Brier score** | **Hosmer–Lemeshow *P*** |
| --- | --- | --- | --- | --- | --- | --- | --- |
| Derivation set (apparent performance) | 483 | 21.1 | 0.847 (0.811–0.883) | — | — | 0.152 | 0.566 |
| Random split validation set | 207 | 21.3 | 0.826 (0.779–0.873) | 0.98 (0.85–1.11) | −0.03 (−0.21 to 0.15) | 0.168 | 0.612 |
| Bootstrap internal validation (1,000 resamples) | 483 (resampled) | — | Optimism‑corrected AUC = **0.831** | — | — | — | — |
| Temporal validation set | 172 | 20.9 | 0.819 (0.768–0.870) | 0.94 (0.79–1.09) | 0.06 (−0.18 to 0.30) | 0.168 | 0.684 |

**Supplementary Figure 1** **Calibration plot of the temporal validation set (n = 172)**
